# Editorial: Novel perspectives and improvements in fMRI functional connectivity analysis methods used to investigate brain networks and cognitive mechanisms in humans

**DOI:** 10.3389/fnins.2023.1222586

**Published:** 2023-05-30

**Authors:** Mete Ozay

**Affiliations:** Samsung R&D Institute UK, Staines, United Kingdom

**Keywords:** fMRI, brain networks, cognitive mechanisms, functional connectivity, brain connectivity

Over the last decade, there has been growing interest and important developments for characterization of the brain connectome to obtain useful and meaningful information while exploring a wide range of pathological conditions and cognitive mechanisms (Bijsterbosch et al., 2021; Bernstein-Eliav and Tavor, 2022; Srivastava et al., 2022). The brain connectome can be investigated using various connectivity measures such as structural (anatomical) and functional (neuronal) connectivity within and between regions in the brain (Babaeeghazvini et al., 2021). Although functional connectivity (FC) can be shaped by structural connectivity (Honey et al., 2010), the interaction between two models may vary depending on the underlying cognitive mechanisms (Litwińczuk et al., 2022; Liu et al., 2023).

In this collection, we present novel perspectives and methods for analysis of functional connectivity modeled by functional MRI (fMRI) techniques which measure localized increase in blood oxygenation levels reflecting the increase in neuronal activity in the brain. The Research Topic “Novel Perspectives and Improvements in fMRI Functional Connectivity Analysis Methods Used to Investigate Brain Networks and Cognitive Mechanisms” consists of a collection of 7 contributions discussing new methods and systems for FC analysis of brain networks to explore the underlying cognitive mechanisms, and report the recent advances in brain research.

The first paper by Wang Q. et al. explores the evolution of intra- and inter-network FC in drug-naive Parkinson's disease (PD) patients with different motor subtypes. This study shows that FC varying among anterior and posterior salience networks can be an imaging marker to distinguish PD with tremor dominant from PD with postural instability and gait disorder. Their methods and results provide a basis that can lead to novel approaches for identification and precision treatment of PD motor subtypes.

In the second paper, Fang et al. first give a detailed review of the related work including applications and toolboxes used for analysis of multivariate connectivity of brain networks. Then, the authors discuss the challenges of modeling non-linear complex neural dynamics of cognitive processes in the brain. Finally, they characterize the steps that can be followed to model non-linear relationships between representations encoded in different brain regions. The proposed steps and open problems can guide researchers to develop novel multivariate connectivity models of non-linear dynamics of the brain.

The third paper by Li et al. investigates changes of brain networks in epilepsy patients without intracranial lesions under resting conditions using graph theoretic methods and methods from statistical signal processing such as independent component analysis (ICA). In the analyses, they observe that the topological characteristics and FC of brain networks are changed in non-lesional epilepsy patients. More precisely, the results suggest that reduced efficiency between brain networks in epilepsy patients and a compensatory response to brain function at earlier stages of the disease can be detected by abnormal FC. Their proposed methods and results can help researchers to identify and explore exclusive brain networks for different types of epilepsy.

In the fourth paper, de Rijk et al. examine homologous clusters in the periaqueductal gray (PAG), which is a brain stem area designated to play an essential role in lower urinary tract (LUT) control, between subjects. To explore the relationship between LUT symptoms, such as urgency, and activity patterns in the PAG in normal and pathological states, they propose employment of various clustering and cluster analysis methods. At the within-subject level, the results suggest that resting-state fMRI data of the PAG can be used to obtain clusters that show some of the anatomical characteristics known from animal and post-mortem studies. At the group level, they observe that PAG clusters additionally show high spatial organizational similarity. Their proposed analyses and findings can help to improve current therapies and patient selection strategies, and lead to the development of new therapies.

The fifth paper by Wang Y. et al. investigates how phase-encoding (PE) direction in echo-planar imaging on gender differences can affect the outcome of a specific research on gender differences. In the analyses, they found that PE direction can partially influence gender differences in spontaneous brain activity of resting-state fMRI. The findings suggest appropriate selection of PE direction as an important criterion to be utilized in resting-state fMRI studies.

In the next paper, Cordes et al. study naturally occurring frequency bands of resting-state data obtained by group ICA using Empirical Mode Decomposition (EMD) which is an adaptive time-frequency method. They show that the intrinsic mode functions of blood-oxygenation level-dependent (BOLD) fMRI signals provide characteristic energy-period profiles that allow a data-driven arrangement of all resting-state networks which is not possible using the non-adaptive methods. The experimental results expand the current understanding of network dynamics in PD. Their proposed novel approach to understanding functional networks in PD, can lead to development of a clinically useful *in vivo* assay of PD network physiology.

In the last paper, Pillet et al. examine the similarities between neural networks derived from representational similarity analysis (RSA) with those from Univariate analysis (UNIVAR) and functional connectivity analysis (FCA) to explore how these methods relate to each other. This work provides first-time evidence that cortical networks derived from three commonly used neuroimaging approaches (RSA, UNIVAR, and FCA) are highly similar regardless of the structural variations of each network. Improving the understanding of the relationship between the functional networks derived from these methods will allow researchers to use these approaches more adequately.

These articles have made outstanding contributions to brain research and applications. We hope that our Research Topic can inspire new studies on the brain analysis leading to further advances in neuroscience using network theory, graph theory, machine learning and statistical signal processing.

## Author contributions

MO has made a substantial, direct, and intellectual contribution to the work and approved it for publication.
